# Artificial intelligence-based automated breast ultrasound radiomics for breast tumor diagnosis and treatment: a narrative review

**DOI:** 10.3389/fonc.2025.1578991

**Published:** 2025-05-08

**Authors:** Yinglin Guo, Ning Li, Chonghui Song, Juan Yang, Yinglan Quan, Hongjiang Zhang

**Affiliations:** ^1^ Faculty of Life Science and Technology & The Affiliated Anning First People’s Hospital, Kunming University of Science and Technology, Kunming, China; ^2^ Department of Radiology, Faculty of Life Science and Technology & The Affiliated Anning First People's Hospital, Kunming University of Science and Technology, Kunming, China

**Keywords:** breast, breast tumor, automatic breast ultrasound, artificial intelligence, radiomics, deep learning, machine learning

## Abstract

Breast cancer (BC) is the most common malignant tumor among women worldwide, posing a substantial threat to their health and overall quality of life. Consequently, for early-stage BC, timely screening, accurate diagnosis, and the development of personalized treatment strategies are crucial for enhancing patient survival rates. Automated Breast Ultrasound (ABUS) addresses the limitations of traditional handheld ultrasound (HHUS), such as operator dependency and inter-observer variability, by providing a more comprehensive and standardized approach to BC detection and diagnosis. Radiomics, an emerging field, focuses on extracting high-dimensional quantitative features from medical imaging data and utilizing them to construct predictive models for disease diagnosis, prognosis, and treatment evaluation. In recent years, the integration of artificial intelligence (AI) with radiomics has significantly enhanced the process of analyzing and extracting meaningful features from large and complex radiomic datasets through the application of machine learning (ML) and deep learning (DL) algorithms. Recently, AI-based ABUS radiomics has demonstrated significant potential in the diagnosis and therapeutic evaluation of BC. However, despite the notable performance and application potential of ML and DL models based on ABUS, the inherent variability in the analyzed data highlights the need for further evaluation of these models to ensure their reliability in clinical applications.

## 1 Introduction

Breast cancer (BC) is one of the most frequent cancers among women worldwide. According to the latest data from the International Agency for Research on Cancer (IARC), approximately 2.3 million new cases of female BC and close to 670,000 related deaths will occur globally in 2022 (1), if it is identified at the early stage, the survival rate is significantly improved.

Automated breast ultrasound (ABUS) is an advanced ultrasound imaging technology approved by the U.S. Food and Drug Administration (FDA) in 2012 (2). Compared with conventional ultrasound, ABUS has several advantages, including operator independence, whole-breast coverage and stable image quality. Additionally, ABUS can reconstruct coronal plane (CP) images, which is not achievable with conventional ultrasound. CP imaging is particularly valuable for the diagnosis of breast lesions as it provides crucial anatomical and symptomatic information (3). Comprehensive analysis of tumors using large volumes of image data from ABUS remains a challenge for sonographers. Additionally, the interpretation of ultrasound images may depend on the clinical experience of sonographers, which can affect the diagnostic accuracy and efficacy of ABUS (4).

Radiomics is a field that analyzes a vast number of medical images to extract numerous features reflecting disease characteristics, and explores the associations between the features and patients’ prognoses for precision medicine (5). Specifically, this technology employs various image processing algorithms to enhance image quality and utilizes diverse techniques and methods for high-throughput data analysis to extract quantitative features—such as shape, texture, and filtering characteristics—from regions of interest (ROIs). Traditional radiomics often uses software to extract and screen those features that can most effectively capture the intra-tumor and inter-tumor heterogeneity. Then, it uses statistical analysis methods such as multivariate logistic regression (LR) analysis to construct the model (6).

Significant advances in the field of Artificial intelligence (AI), it holds promise in increasing the diagnostic value of ultrasound imaging for histological analysis based on machine learning (ML) and deep learning (DL) (7, 8). Radiomics can enhance the utility of existing data for clinicians by integrating advanced mathematical analysis from AI (9). The integration of radiomics with imaging tool like Magnetic resonance imaging (MRI) and mammography (MG) shows promise for early BC screening (10, 11). In breast ultrasonography, the large volume of data, easy accessibility of images, and diverse image types have led to the increasing involvement of AI-based radiomics in the diagnostic process. Currently, multimodal ultrasound radiomics is one of the most active areas of investigation (12–14). ABUS, an emerging breast imaging technology, offers high-quality image presentation, allowing for the extraction of more precise ultrasound features with strong diagnostic potential.

We searched on PubMed and Web of Science for publications from 2020 up to September 2024. The search keywords included “Automated Breast Ultrasound”, “Breast Cancer”, “Deep Learning”, “Machine Learning” and “Artificial Intelligence”. A total of 46 relevant papers were included. This review outlines the fundamental concepts of ABUS, radiomics, and AI, aiming to summarize the current status and research progress of ABUS radiomics based on traditional ML and DL algorithms in the applications of assisting BC detection, diagnosis, classification, and prognosis evaluation.

## 2 Methods

**Figure 1:**
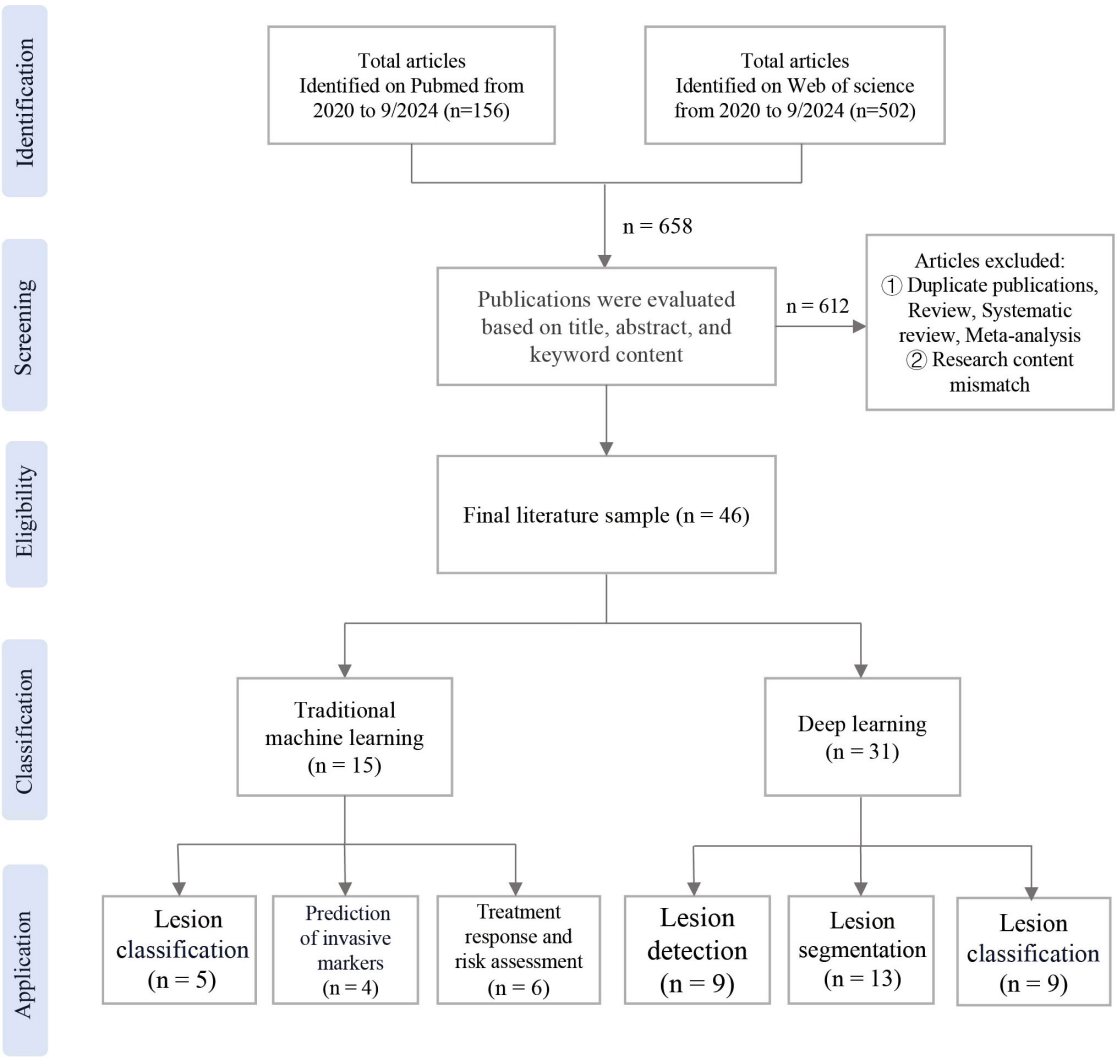
Flow diagram of study selection.

The publications were searched in the databases of PubMed and Web of Science. The search was limited to studies published between 2020 and September 2024. Exclusion criteria were applied, which were not associated with radiomics. Additionally, studies consisting solely of review, systematic review, meta-analysis were excluded. Following the removal of duplicate studies, A total of 46 relevant papers were included, the workflow of the study was shown in **Figure 1**.

## 3 Introduction to ABUS and radiomics

### 3.1 Overview and workflow of ABUS

Studies have shown that women with extremely dense breast tissue have a 4.7 times higher risk of breast cancer compared to those with lower breast density (15). Since the 1980s, researchers have proposed the development of ABUS technology in response to the limitations of MG in screening dense breasts (16, 17). Currently, ABUS has been evaluated as a complementary tool to MG, providing the ability to scan the entire breast without operator variability while maintaining the benefits of handheld ultrasound (HHUS), including superior tissue penetration and lesion characterization (18).

**Figure 2:**
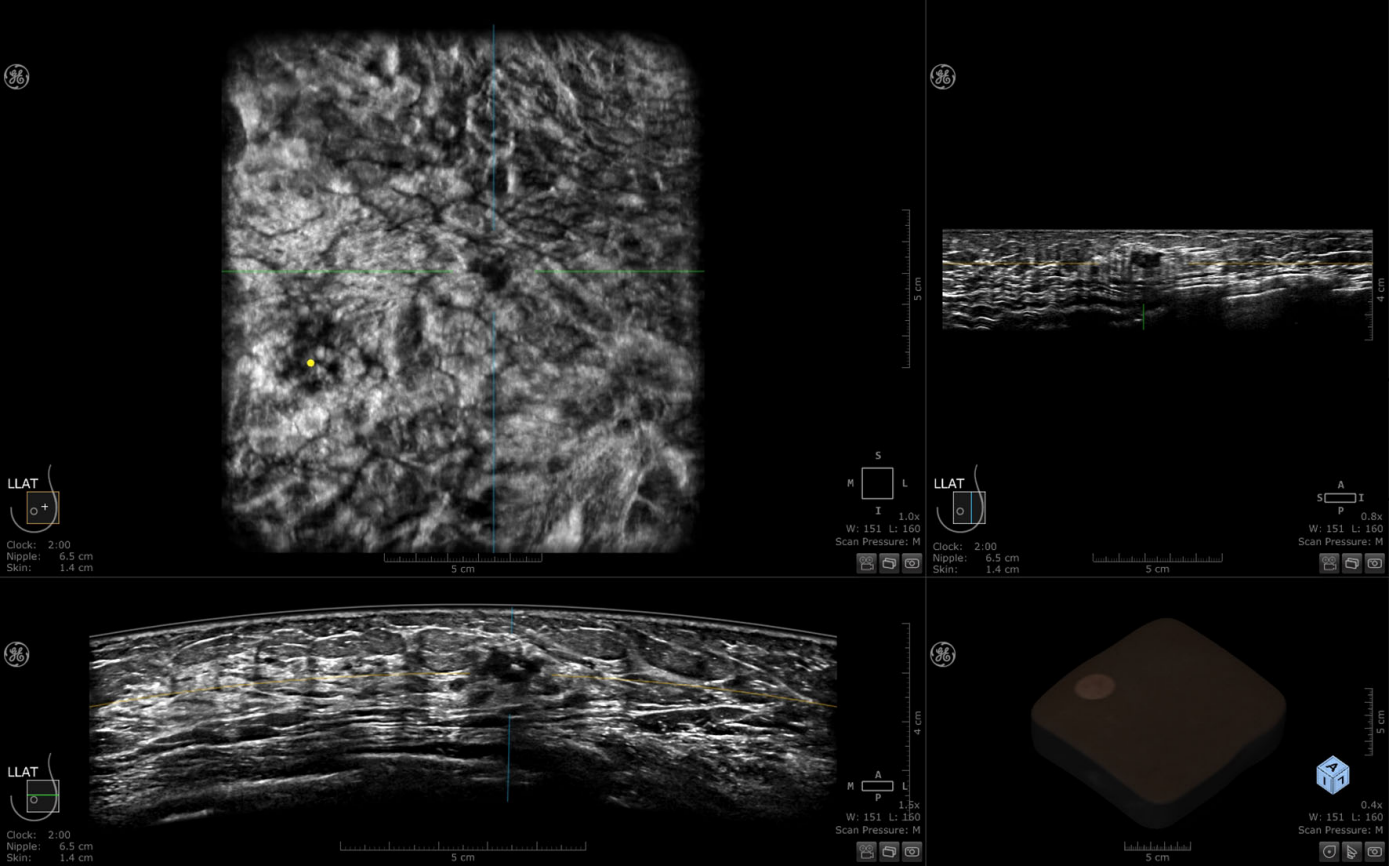
A set of 3-D ABUS images generated by Invenia ABUS.

ABUS is a computer-based ultrasound screening system designed to evaluate the entire breast tissue using an automated probe. This system ensures symmetry and bilaterality in screening results. Each breast is imaged in three different views: axial, sagittal, and coronal. Volumetric data is stored and transferred to a workstation after the scan. This eliminates the need for sonographers to perform image acquisition and interpretation simultaneously during breast screening using ABUS. ABUS helps standardize breast ultrasound and overcome limitations of ultrasound, including reduced operator dependency and shorter examination times (3). Currently available ABUS systems include both prone and supine scanning modes (19), the most commonly used in clinical practice are mainly supine, including the Invenia ABUS system from GE and ACUSON S2000 automated breast volume scanner (ABVS) from Siemens (3, 20). In this article, the 3D Automated Breast Ultrasound technology is uniformly referred to as ABUS. The basic structure of both ABUS systems includes a main unit (ultrasound diagnostic device), high-frequency and large-sized sensors, a curved transducer, and an image data processing system. A set of ABUS images generated by Invenia ABUS is displayed in **Figure 2**.

### 3.2 Radiomics

Computer-Aided Detection (CAD) has greatly enhanced disease detection and diagnosis by extracting tumor characteristics through advanced computational analysis, particularly in breast and lung cancer screening. Recently, radiomics has emerged as an important extension of CAD (21), which involves extracting numerous quantitative features from medical images using automated or semi-automated high-throughput software tailored to a specific imaging modality. This process identifies relevant features that reflect both macroscopic (e.g., tumor shape and texture) and microscopic information (e.g., pathology and genetics), enabling predictive models for clinical functions such as screening, diagnosis, prognosis, and efficacy assessment (22, 23).

Currently, ultrasound-based radiomics employs two distinct approaches for feature extraction: semi-automatic methods requiring manual segmentation with subsequent traditional ML analysis (24), and fully automated DL methods capable of performing end-to-end tasks including image segmentation, lesion detection, and classification. These DL techniques can be further categorized into supervised, unsupervised, and semi-supervised learning paradigms (25, 26).

**Figure 3:**
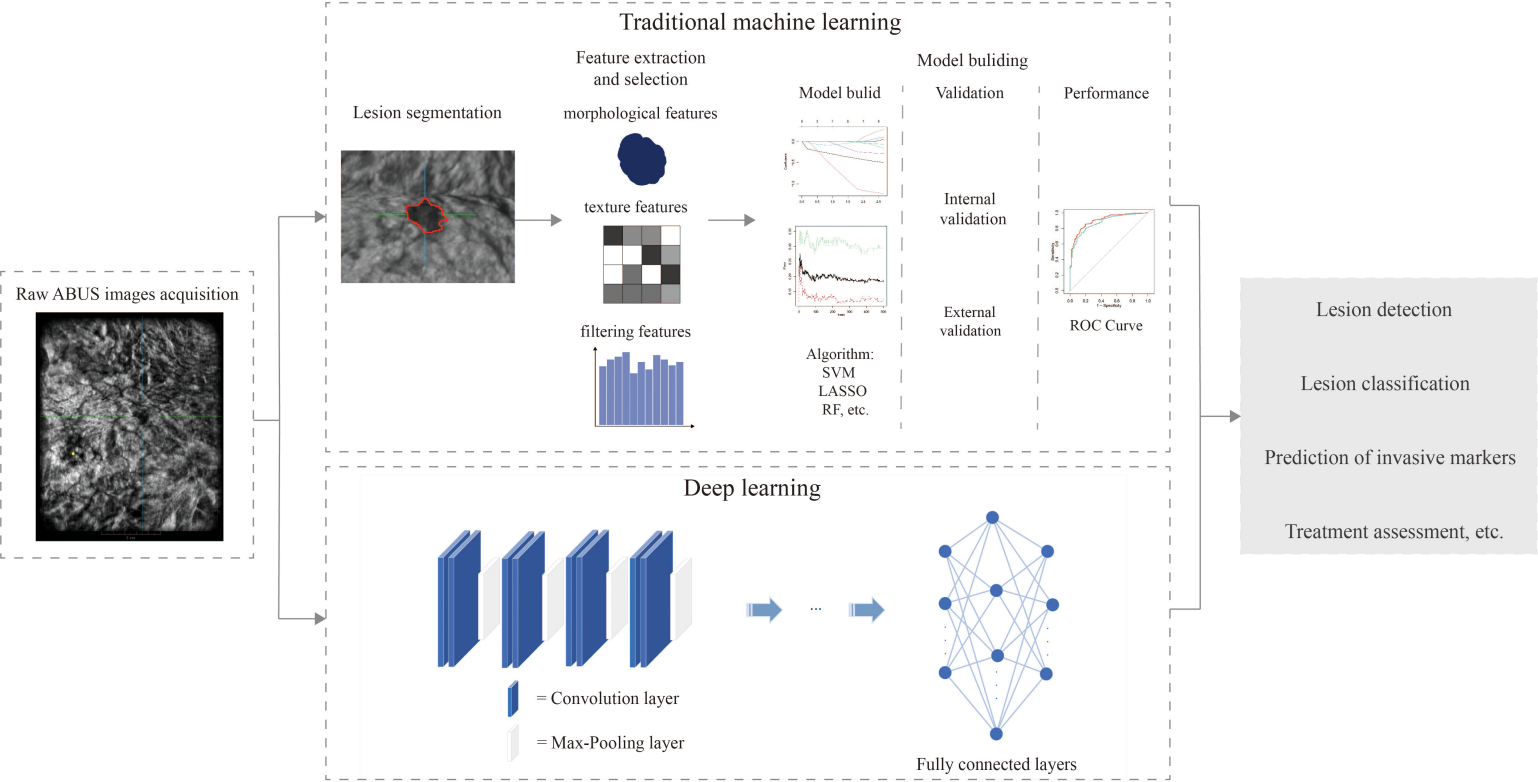
The radiomics workflow for ABUS imaging integrates two distinct methodologies. Traditional machine learning relies on manual processes including image acquisition, feature extraction and selection, followed by model construction to complete tasks. Deep learning primarily employs convolutional neural networks (CNN) composed of three core components: convolutional layers for local feature extraction, pooling layers for dimensionality reduction, and fully connected layers that map extracted features to output layers for final task execution. SVM, Support Vector Machine; LASSO, Least Absolute Shrinkage and Selection Operator; RF, Random Forest; ROC, Receiver Operating Characteristic.

The current workflow of radiomics is divided into five main steps: ①Image acquisition. In clinical practice for BC, commonly used imaging modalities include ultrasound, MG, and MRI. Medical images must be acquired in strict compliance with operational standards (27). ②Pre-processing and tumor segmentation. Image pre-processing typically involves noise removal and resolution enhancement. Because of the lack of standardized methods for segmentation and feature extraction, image segmentation primarily focuses on delineating the ROIs (28). Common image segmentation techniques include manual, semi-automatic, and automatic segmentation (29). ③Feature Extraction, which includes statistical texture features, morphological features, and filtering features. DL extracts more abstract features directly from raw data (27). ④Feature selection to reduce overfitting by using methods like filtering methods (23), wrapper methods, and embedding methods (30). ⑤Modeling to predict disease prognosis and biological behavior through feature analysis, followed by training ML or DL models, the models were evaluated through cross-validation and external validation sets (23, 27, 31). The ABUS radiomics workflow integrating both traditional ML and DL approaches is shown in **Figure 3**.

## 4 Introduction and applications of traditional machine learning for ABUS radiomics

### 4.1 Introduction of traditional machine learning for ABUS radiomics

ML is a critical branch of AI that leverages datasets to train models and generates predictions by synthesizing information from all data samples. These predictions can then be used to assist in clinical decision-making. As of November 2022, the FDA has approved 521 ML algorithms for clinical use, of which 392 can be applied to radiomics (32). To enable clinical applications, common ML methods can be categorized into the following types, including reinforcement learning, unsupervised learning, and supervised learning (33). ML algorithms used in traditional radiomics are typically supervised. Supervised learning involves training a model on labeled data, allowing the model to learn the mapping between inputs and outputs with instances of the expected outputs labeled by a human which is referred to as the “ground truth” (34).

In ABUS radiomics, traditional ML algorithms are mainly applied to texture analysis, screening, and extraction of quantitative features from radiomics images to create predictive models and decision-support tools. The steps of ultrasound radiomics based on traditional ML algorithms are mainly divided into image segmentation, feature extraction, feature screening, and model build. The image segmentation part is mainly performed in a semi-automatic form, with one or more physicians with years of imaging experience performing ROIs regions through platforms such as 3D Slicer, ITKSNAP, MaZda, SEG3D2, and Deepwise. The manual delineation of ROIs is a labor-intensive and time-consuming process (35–39). The Python-based *pyradiomics* package is a widely used tool for feature extraction in radiomics studies. It provides a convenient and comprehensive open-source platform that can efficiently process and extract numerous radiomic features (40, 41). For feature selection, statistical tests such as the t-test, chi-square test, Least Absolute Shrinkage and Selection Operator (LASSO) regression, and Gradient Boosted Decision Trees (GBDT) are commonly employed to reduce the feature dimensionality and enhance the model’s generalization ability.

Although traditional radiomics methods require manual feature extraction, many clinicians prefer these approaches for constructing models and implementing clinical functions. This is because of their advantages in terms of model interpretability and flexibility in clinical applications. These methods have been effectively used to differentiate between benign and malignant tumors, predict axillary lymph node metastasis, assess the efficacy of neoadjuvant therapies, and predict preoperative Ki-67 expression levels. **Table 1** summarizes the performance of traditional ML methods applied to ABUS radiomics.

**Table 1:** The performance of traditional ML methods applied to ABUS radiomics.

Year	Author	Classifier	Device	Image modalities	No. of patients	Performance	Description	Study design	Training targets
2022	Wang et al. (42)	SVM, LR, XGBoost	Simens Acuson S2000	ABVS	200	AUC= 0.857 ± 0.058, SEN=87.9%, SPE=68.2%, ACC=80.7%, PRE=82.9%	Developed a radiomics model by extracting 208 features from multi-planar ABVS images, selecting features using RFE, RF, and chi-square tests, and evaluating classifiers SVM, LR, XGBoost with 5-fold cross-validation	Single-center study; Internal validation	Classification: malignant vs. benign
2022	Wang et al. (39)	LR	Simens Acuson S2000	ABVS	193	Training set: AUC= 0.959, ACC= 91.6%, SEN=90.2%,SPE= 92.7%,PPV= 91.4%, NPV=91.8% Validation set: AUC= 0.925, ACC= 86.7%, SEN=83.3%,SPE= 90.5%,PPV= 90.9%, NPV=82.6%	Developed a radiomic nomogram by extracting 1101 features from multi-planar ABVS images, selecting 19 key features through ICC > 0.75, mRMR and LASSO, and combining them with clinical factors (lesion size, BI-RADS 4 subcategories) via LR	Single-center study; Internal validation	Classification: malignant vs. benign
2023	Ma et al. (36)	LR	Simens Acuson S2000	ABVS, SE, US	620	Training set: AUC= 0.975, ACC= 93.78%, SPE= 92.02%, SEN= 96.49% Validation set: AUC= 0.946, ACC= 87.63%, SPE= 83.93%, SEN= 93.24%	Developed a multimodal radiomics model by extracting 1652 features from ABVS, B-ultrasound, and SE, selecting 14 key features through ICC > 0.75, univariate correlation analysis, GBDT, and combining selected features through multivariate LR	Single-center study; Internal validation	Classification: malignant vs. benign
2023	Guo et al. (43)	LR	Simens Acuson S2000	ABVS, SE	423	Training set: AUC=0.972 Validation set: AUC=0.964	Developed a clinical-radiomics nomogram by extracting radiomics features from ABVS and SE images, screening features using Mann-Whitney U test, and LASSO with tenfold cross-validation, and combining selected features with clinical risk factors and BI-RADS scores through multivariate LR	Single-center study; Internal validation	Classification: malignant vs. benign
2023	Ma et al. (44)	GBDT, LR	Simens Acuson S2000	ABVS, US	190	Training set: AUC= 0.900, ACC= 80.9%, SPE= 79.5%, SEN= 85% Validation set: AUC= 0.911, ACC= 86.8%, SPE= 89.3%, SEN= 80%	Developed a radiomics nomogram by extracting features from ABVS and US images, screening them using univariate correlation analysis and GBDT, then combining selected radiomics features with clinical factors (age, VTI score, SWV) through multivariate LR	Single-center study; Internal validation	Classification: malignant vs. benign
2023	Li et al. (45)	LR, SVM, RF, XGBoost, KNN	GE Healthcare Invenia ABUS	ABUS	936	Training set: AUC=0.868 Testing set: AUC=0.822	Developed a predictive model by extracting 2632 radiomics features from intratumoral and peritumoral regions in ABUS images, selecting 15 key features through Z-score normalization, ICC, Wilcoxon rank-sum test, mRMR, and LASSO regression, and integrating them via SVM classifier	Dual-center study; Internal validation	Predict Ki-67 expression patterns
2024	Wu et al. (46)	LR	Simens Acuson S2000	ABVS	197	Training set: AUC=0.905, SPE= 0.944, SEN=0.745 Validation set: AUC=0.882, SPE=0.909, SEN=0.727	Developed a radiomics nomogram by extracting 1702 features from intratumoral and peritumoral regions in ABVS images, selecting 15 key features using LASSO regression, and integrating them with ultrasound-reported lymph node status and tumor size through multivariate LR	Single-center study; Internal validation	Predict Ki-67 expression patterns
2024	Wang et al. (35)	RF, LGBM, GBC, ETC	Simens Acuson S2000	ABVS	271	Validation set: AUC=0.826, SPE= 0.750, SEN=0.909 Test set: AUC=0.700, SPE=0.419, SEN=0.861	Developed a radiomics model by extracting features from intratumoral and peritumoral regions on ABVS images, combined with clinical and serological features, and employed model-weighted ensemble methods to predict HER2 status, with feature selection performed using classifiers like RF, LGBM, GBC, ETC	Multi-center study; External validation	Preoperative prediction of HER2 status
2024	Hu et al. (47)	SVM, RF, XGBoost	Simens Acuson Oxana 2	ABVS	668	Training set: AUC=0.957 Testing set: AUC=0.920, ACC= 0.846, PRE=0.907	Developed a predictive model by extracting 1409 radiomics features from intratumoral and peritumoral regions in ABVS images, selecting key features using ICC, WRS, mRMR, and LASSO regression, and integrating them through XGBoost classifier	Dual-center study; External validation	Predict Ki-67 expression patterns
2023	Jiang et al. (48)	LR, SVM, RF	Simens Acuson Oxana 2	ABVS	248	Pre-NAC: AUC=0.790, SEN=0.364, SPE=0.821, ACC=0.620 Post-NAC: AUC=0.890, SEN=0.500, SPE=0.929, ACC=0.773	developed an ABVS-based radiomics model by extracting 2628 features from pre- and post-NAC ultrasound images, selecting 13 key features using LASSO regression, and evaluating their predictive performance for NAC response using LR, SVM, and RF classifiers with five-fold cross-validation	Single-center study; Internal validation	Predicting the Efficacy of Neoadjuvant Chemotherapy
2023	Chen et al. (49)	LR	GE Healthcare Invenia ABUS	ABUS	310	Training set: AUC=0.876, SEN=0.853, SPE=0.826, ACC=0.831, PPV=0.538, NPV=0.959 Test set: AUC=0.851, SEN=0.765, SPE=0.825, ACC=0.814, PPV=0.481, NPV=0.943	Developed a radiomics nomogram by extracting 837 features, selecting 13 key features using LASSO with ten-fold cross-validation, and integrating them with retraction phenomenon and US-reported ALN status through multivariate LR	Single-center study; Internal validation	Predicting axillary lymph node tumor burden
2023	Li et al. (50)	SVM, LR	Simens Acuson S2000	ABVS	335	Training set: AUC=0.950, SEN= 83.67%, SPE= 92.47%, ACC= 90.64%, PPV= 74.55%, NPV= 95.56% Validation set: AUC=0.88, SEN= 70.00%, SPE= 88.75%, ACC= 85.00%, PPV= 60.87%, NPV= 92.21%	Developed an ABVS-based radiomics model by extracting 5901 features, selecting key features per plane using LASSO regression with ten-fold cross-validation, and integrating them with convergence sign, strain elasticity level, positive SLN number and Ki67 index through LR	Single-center study; Internal validation	Prediction of lymphovascular invasion status
2023	Li et al. (38)	LR	GE Healthcare Invenia ABUS	ABUS	517	Training set: AUC=0.924 Validation set: AUC=0.812	Developed a radiomics nomogram by first extracting 1316 radiomics features from ABUS images, selecting 1109 features with ICCs ≥ 0.8, then identifying 7 key features via Student’s t-test and LASSO regression, which was combined with tumor size, US-reported LN status, and ABUS retraction phenomenon in LR to build the predictive model	Multi-center study; External validation	Prediction of metastatic lymph node burden
2023	Wang et al. (51)	LR	Simens Acuson S2000	ABVS, US	276	Training set: AUC= 0.781, SEN=0.838, SPE=0.634, ACC=0.722 Validation set: AUC= 0.773, SEN=0.800, SPE=0.800, ACC=0.800 Test set: AUC= 0.828, SEN=0.853, SPE=0.660, ACC=0.742	Developed an ABVS-based radiomics nomogram by extracting 110 features, selecting key features through LASSO regression with ten-fold cross-validation (ICC ≥0.80), and integrating them via multivariate LR	Multi-center study; External validation	Assessment of axillary lymph node metastasis risk
2024	Hu et al. (37)	LR	Simens Acuson Oxana 2	ABVS	511	Training set: AUC= 0.910 Validation set: AUC= 0.882	Developed an ABVS-based radiomics nomogram by extracting 1116 features, selecting 11 key features through LASSO regression with ten-fold cross-validation (ICC ≥0.80), and integrating them with clinical predictors via multivariate LR	Multi-center study; External validation	Predicting ≤3 positive axillary lymph nodes in HR+/HER2- breast cancer with 1–2 positive sentinel nodes

SVM, support vector machine; LR, logistic regression; XGBoost, extreme gradient boosting; RFE, recursive feature elimination; RF, random forests; ICC, intraclass correlation coefficient; mRMR, minimum redundancy maximum relevance; SE, Strain elastography; US, ultrasound; GBDT, Gradient Boosted Decision Trees; SWV, shear wave velocity; LASSO, least absolute shrinkage and selection operator; RF, random forests; KNN, k-nearest neighbors; LGBM, Light Gradient Boosting Machine; GBC, Gradient Boosting Classifier; ETC, Extra Tree Classifier; AUC, Area Under the Curve; SEN, sensitivity; SPE, specificity; ACC, accuracy; PRE, precision; PPV, positive predictive value; NPV, negative predictive value; WRS, Wilcoxon rank sum.

### 4.2 Applications of traditional machine learning for ABUS radiomics

#### 4.2.1 Lesion classification

Accurate screening of benign and malignant breast lesions enables timely intervention in patients with malignant tumors, significantly improving survival rates and preventing unnecessary treatments for those with benign lesions. Currently, the accurate and consistent identification of certain lesions through visual inspection remains challenging. Several studies have explored the diagnostic potential of traditional ML techniques applied to ABUS radiomics to distinguish between benign and malignant breast lesions. ABUS, as a 3D ultrasound modality, provides a three-plane view, with image segmentation typically performed using axial plane (AP) and CP. Images obtained from these two planes are generally easier to analyze and interpret. This aligns better with the reading preferences of radiologists and the requirements of clinical examination.

Wang et al. manually segmented the largest lesions in AP and CP views using ITK-SNAP and developed a classification model with Support Vector Machine (SVM), Logistic Regression (LR), and Extreme Gradient Boosting (XGBoost). The SVM model achieved the best performance, with an Area Under the Curve (AUC) of 0.857 ± 0.058 and sensitivity (SEN), specificity (SPE), accuracy (ACC), and precision (PRE) values of 87.9%, 68.2%, 80.7%, and 82.9%, respectively. The study found that features from the AP provided superior classification performance compared to the CP, and leveraging data from both planes enabled the construction of a model with more comprehensive performance (42).

Ma et al. developed an ABVS model using CP features and a multimodal model combining ABVS, B-ultrasound, and strain elastography (SE). The multimodal model demonstrated superior performance compared to other models, achieving an AUC of 0.946 on the validation set, significantly enhancing the model’s diagnostic efficacy. Several studies have demonstrated that multimodal models exhibit superior performance compared to unimodal imaging models (36, 43). Multimodal models involving ABUS data are typically based on combinations of ABUS with HHUS, SE, contrast-enhanced ultrasound (CEU), or digital breast tomosynthesis (DBT) (43, 44, 52). Since ABUS provides three-dimensional views, multiplanar features can significantly improve classification performance. Compared to HHUS, ABUS’s CP texture features demonstrate superior performance in distinguishing benign and malignant lesions. However, reconstructed coronal images have relatively lower resolution, which may lead to the omission of critical features such as microcalcifications. Additionally, most current studies focus on features extracted from the largest cross-sectional area of tumors, leaving the three-dimensional volumetric information underutilized. Future research should explore dynamic imaging characteristics of benign and malignant lesions to further enhance diagnostic accuracy.

#### 4.2.2 Prediction of invasive markers

BC is a highly heterogeneous disease, with multiple potential therapeutic molecular targets that play crucial roles in cell metastasis, invasion, apoptosis, and cell cycle regulation. Ki-67 is a crucial biomarker for assessing BC; its expression level reflects the tumor’s proliferative activity (53, 54). Accurate Ki-67 assessment provides valuable information for BC management (45–47).

Wu et al. developed an ABVS-based radiomics model for Ki-67 prediction by analyzing ROIs in CP across different Ki-67 expression levels. They reconstructed peritumoral ROIs using a dilation algorithm, selected features through LASSO regression, and constructed the predictive model via LR. The model demonstrated robust performance with AUC values of 0.905 in the training set and 0.882 in the validation set. Utilizing readily accessible input data, this approach shows significant clinical value (46).

The expression of human epidermal growth factor receptor 2 (HER2) is closely linked to the prognosis of BC, with HER2-positive cases accounting for about 14% of all female BC cases (55). These patients demonstrate greater heterogeneity, and lower survival rates. HER2 expression indicates clinical aggressiveness and aids physicians in treatment decisions (56, 57). Since trastuzumab received approval for HER2-positive metastatic BC in 1998, several tyrosine kinase inhibitors (TKIs) and antibody-drug conjugates (ADCs) targeting HER2 have been approved for clinical use (58). Due to its critical role, HER2 has become an essential diagnostic and therapeutic biomarker for BC (59).

Wang et al. developed four optimal models using weighted and feature combination methods to predict HER2 status in BC using ABVS-based radiomics features. The weighted combination model achieved an AUC of 0.700, SEN of 86.1%, and SPE of 41.9% in test set (35). Due to the lack of a strict standard for ROIs division, outlining accuracy can impact model performance. To minimize operator bias in semi-automatic segmentation, multiple sonographers are employed for ROIs delineation. Optimized weighted combination models that include ABVS-based intra tumoral and peritumoral radiomics features along with clinical data show promise for noninvasive preoperative prediction of HER2 status in BC.

However, current ABUS primarily provides morphological and echogenic features while lacking functional information (e.g., hemodynamics), which may limit its predictive capability for certain biomarkers. Therefore, multimodal integration becomes particularly crucial for improving the accuracy of predicting tumor marker expression levels.

#### 4.2.3 Treatment response and risk assessment

Neoadjuvant therapy (NAT) is a common treatment for early-stage BC that reduces tumor size, enhances surgical removal success, and increases the likelihood of breast-conserving surgery (60, 61). It can also eliminate axillary lymph node metastases (ALND) detectable during sentinel lymph nodes (LNs) biopsy post-treatment. Currently, effectiveness assessments mainly rely on post-treatment evaluations, which may delay timely treatment adjustments.

Jiang et al. collected ABVS images from patients within one week before the start of NAC and one week after the second NAC cycle to identify image features associated with NAT efficacy. The results showed that the prediction performance of features extracted after the second treatment cycle was significantly superior to that of pretreatment features (AUC 0.89 vs. 0.79). This suggests that postoperative assessment differs from pre-treatment evaluation, likely due to changes in the tumor microenvironment following treatment (48).

ALND, including the number and distribution of positive LNs, is a key factor in determining the pathological stage of BC (62). Therefore, an accurate assessment of ALND involvement is essential for developing appropriate treatment plans. ALND can be diagnosed by sentinel lymph node biopsy in patients with early-stage BC (63), the false-positive rate is approximately 10% (64); therefore, developing a radiomics model for the assessment of ALND is clinically significant. Chen et al. compared several models, including an ABUS feature model with tumor diameter, retraction phenomenon, hyperechoic halo, ABUS radiomics model, and multi-modal ABUS radiomics model incorporating ultrasound reports of axillary lymph node status and retraction phenomenon. The AUC value for the training set was 0.876 and the test set was 0.851 (49). Current research primarily focuses on the extraction and analysis of radiomic features. However, the complexity of the tumor microenvironment suggests that predicting LNs should not rely solely on imaging data. Given that tumor invasiveness, immune microenvironment status, and molecular heterogeneity all influence metastatic risk, future studies should integrate multimodal data, including pathological characteristics and molecular biomarkers, to develop a comprehensive “imaging-pathology-molecular” predictive model.

## 5 Introduction and applications of deep learning for ABUS radiomics

### 5.1 Introduction of deep learning for ABUS radiomics

Since AlexNet demonstrated its remarkable performance in image recognition challenges, DL, as a key branch of AI, has gained widespread attention (65). DL is a crucial subfield of ML that focuses on representation learning through hierarchical nonlinear transformations. It is particularly adept at processing unstructured data (e.g., images, text) and can adapt to supervised, unsupervised, or semi-supervised learning paradigms (66, 67). This is achieved by connecting simple nonlinear modules through a multilayered neural network that mimics the structure of the human brain (68). The emergence of DL has expanded the range of applications in the field of computer vision (67). In imaging, DL employs multilayer neural networks to transform input data into outputs that align with desired outcomes. Common types of outputs include object locations for lesion detection, pixel labels for image segmentation, and image categories for lesion classification. The basic architecture of DL is Convolutional Neural Network (CNN) (69). Inspired by the biological visual cortex, a CNN typically comprises three primary components: convolutional layers, pooling layers, and fully-connected layers (70). The convolutional layers serve as the core feature extractors, applying multiple convolutional kernels to capture local patterns like edges and textures. Pooling layers then reduce spatial dimensionality while preserving critical features. These extracted features are subsequently mapped to outputs through fully-connected layers for final classification or prediction tasks. As the network depth increases, CNNs progressively learn more complex hierarchical representations (68). As combinations of layers have become more diverse, deep neural network architectures built on CNN have been successfully applied to image analysis. Notable examples include AlexNet (65), VGGNet (71), ResNet (72), DenseNet (73), etc. In recent years, the establishment of several medical imaging databases has facilitated data mining and the development of high-performance models has been significantly simplified by the widespread use of generalized neural network frameworks and automated processing workflows (74). Meanwhile, Transformer architectures based on self-attention mechanisms have also demonstrated promising potential in medical image analysis, significantly improving the accuracy of lesion classification and segmentation through their superior long-range dependency modeling capabilities (75).

Currently, DL, one of the most powerful data-driven AI technologies, enables the development of fully automated workflow. DL models generally achieve higher accuracy and performance than traditional ML algorithms. As the field of DL continues to evolve, these algorithms are expected to become mainstream tools for medical radiomics in the future. To date, DL algorithms have been applied to ABUS radiomics for tasks such as lesion detection, tumor segmentation, and lesion classification. **Table 2** summarizes the performance of DL methods applied to ABUS radiomics.

**Table 2:** The performance of DL methods applied to ABUS radiomics.

Year	Author	Methods	Device	Image modalities	No. of patients	Performance	Description	Study design	Training targets
2020	Wang et al. (76)	3D CNN	GE Healthcare Invenia ABUS	ABUS	363	SEN of 95% with 0.84 FPs/volume	The study proposes a 3D CNN with threshold loss, integrating multi-layer feature supervision and adaptive voxel-wise thresholding for lesions detection	Single-center study; Internal validation	lesions detection
2020	Wang et al. (77)	3D U-Net	GE Healthcare Invenia ABUS	ABUS	264	SEN of 91% with 1.92 FPs/volume	The study proposes a 3D U-Net with spatial attention and residual blocks for lesions detection	Single-center study; Internal validation	lesions detection
2020	Moon et al. (78)	3D CNN	GE Healthcare Invenia ABUS	ABUS	246	Test set: SEN of 100% (81/81), 95.3% (77/81), and 90.9% (74/81) with the FPs/per case of 21.6, 6.0, and 4.6	The study proposes a 3D CNN with ensemble learning and focal loss for tumor detection	Single-center study; Internal validation	lesions detection
2020	Wang et al. (79)	3D Inception Unet	GE Healthcare Invenia ABUS	ABUS	196	SEN of 95.1% with 3.0 FPs/volume (abnormal) and 1.3 FPs/volume (normal), IoU of 60.8%, and CenDis of 2.5 mm	The study proposes a 3D Inception U-net with an asymmetric loss function to maintain low false positives	Single-center study; Internal validation	lesions detection
2020	Li et al. (80)	YOLOv3	Simens Acuson S2000	ABUS	124	SEN of 90%, 85%, 80%, 75% and 70% at 7.42, 3.31, 1.62, 1.23 and 0.88 FPs/volume	The study proposes an improved YOLOv3 network for 2D slice detection with a rescoring algorithm and tubelet model to enhance spatial consistency and reduce false positives	Single-center study; Internal validation	lesions detection
2022	Luo et al. (81)	3D U-Net	GE Healthcare Invenia ABUS	ABUS	397	Training set: SEN of 93.8% (volume-based), 97.2% (lesion-based), and 100% (patient-based) with 1.9 FPs/volume Test set: 92.7%, 94.5%, and 96.5% with 2.4 FPs/volume	The study proposes an optimized 3D U-net with densely deep supervision and threshold mapping for lesion detection	Single-center study; Internal validation	lesions detection
2023	Oh et al. (82)	Faster R-CNN, U-Net	GE Healthcare Invenia ABUS 2.0	ABUS	131	test data at best: SEN of 93.65% with 8.6 FPs	The study proposes Faster R-CNN on transverse, coronal, and sagittal ABUS images, followed by hierarchical clustering and U-Net-based postprocessing to reduce false positives	Single-center study; External validation	lesions detection
2023	Li et al. (83)	SSL-E	Simens Acuson S2000	ABUS	124	SEN of 90.2% with 0.15 FPs/image	The study proposes an SSL-E model combining self-training with pseudo-labels to consist regularization, and a copy-paste strategy to enhance tumor diversity and address class imbalance	Single-center study; Internal validation	lesions detection
2023	Malekmohammadi et al. (84)	Patch Bi-ConvLSTM	Siemens ACUSON S2000 and U-system SomoVu ScanStation	ABUS	43	SEN of 82% with 2 FPs/volume	The study proposes a patch-based Bi-ConvLSTM network by leveraging inter-slice correlations and generating heat maps for localization	Multi-center study; Internal validation	lesions detection
2020	Lei et al. (85)	Mask Scoring R-CNN	GE Healthcare Invenia ABUS	ABUS	70	Test set: DSC= 82.1% ± 14.5%, JAC= 71.6% ± 16.2%, HD95 = 1.66 ± 1.13 mm, MSD= 0.48 ± 0.37 mm, RMSD= 0.75 ± 0.51 mm, CMD= 0.67 ± 0.73 mm	The study developed a Mask scoring R-CNN with an attention gate and mask score head for tumor segmentation	Multi-center study; External validation	lesions segmentation
2020	Pan et al. (86)	SC-FCN-BLSTM	Simens Acuson S2000	ABUS	124	DSC=0.8178, REC=0.8067, PRE=0.8292, HD95 = 11.1367	The study proposed an SC-FCN-BLSTM network that integrates bidirectional LSTM to model inter-slice dependencies and spatial-channel attention to enhance discriminative feature fusion	Single-center study; Internal validation	lesions segmentation
2021	Zhou et al. (87)	Encoder-decoder network	Simens Acuson S2000	ABUS	107	DSC=0.778 ± 0.145, JI=0.650 ± 0.170, HD95 = 3.303 ± 4.513mm	The study proposed an encoder-decoder network with iterative feature refinement to improve boundary delineation for tumor segmentation	Single-center study; Internal validation	lesions segmentation
2022	Cao et al. (88)	Auto-DenseUNet	Simens Acuson S2000	ABUS	107	Test set: DSC=77.8% ± 10.3%, JI=64.6% ± 12.4%, HD=4.63 ± 2.41 mm, PRE=80.2% ± 14.5%, REC=78.2% ± 12.4%	The study proposes an Auto-DenseUNet framework utilizing neural architecture search with densely connected structures and multiscale aggregation nodes for tumor segmentation	Single-center study; External validation	Searchable neural network architecture for mass segmentation
2022	Cheng et al. (89)	DSGMFFN	Simens Acuson S2000	ABUS	107	DSC=84.54%, IoU=73.24%, ACC= 99.03%, PRE= 80.15%	The study proposes a DSGMFFN framework integrating deepest semantic guidance in the decoder, multi-scale feature fusion, and a mixed self-attention mechanism for tumor segmentation	Single-center study; External validation	lesions segmentation
2022	Wang et al. (90)	ResNet	GE Healthcare Invenia ABUS	ABUS	743	AUC=0.85, (average precision) AP=0.9, SEN= 85.00%, SPE= 66.67%, F1 scores = 0.82	The study proposes a novel deep learning network integrating an automatic segmentation network with ResNet architectures to enhance morphological feature extraction	Single-center study; Internal validation	lesions segmentation
2022	Zhou et al. (91)	3D Mask R-CNN, VNet	Simens Acuson S2000	ABUS	107	DSC= 64.57% ± 29.13, JC= 53.39% ± 26.87, REC= 64.43% ± 31.89, PRE= 74.51% ± 25.91, 95HD= 11.01 mm ± 10.87, ASD= 4.63 mm ± 8.60	The study proposed a cross-model attention-guided network integrating V-Net and 3D Mask R-CNN with a hybrid loss to enhance tumor segmentation	Single-center study; Internal validation	lesions segmentation
2023	Pan et al. (92)	3D U-Net	Simens Acuson S2000	ABUS	107	After distilling knowledge from the teacher network (3D U-Net), the DSC of the student network (small 3D U-Net) is improved by 7%. Moreover, the DSC of the student network (3D HR-Net) reaches 0.780, which is very close to that of the teacher network, while their parameters are only 6.8% and 12.1% of 3D U-Net	The study proposed a knowledge distillation method with decoupled contrastive learning and ranking loss to enhance ABUS tumor segmentation by efficiently transferring teacher network knowledge to a lightweight student network	Single-center study; Internal validation	lesions segmentation
2023	Li et al. (93)	MDCCM	Simens Acuson S2000	ABUS	107	the private ABUS dataset: DSC= 66.77% 95HD=9.33mm the public dataset: DSC= 62.13% 95HD=11.95mm	The study proposed a generative adversarial network with multi-domain consistency constraints to enhance semi-supervised lesion segmentation by leveraging labeled and unlabeled data through adversarial learning and geometric constraints	Multi-center study; External validation	lesions segmentation
2024	Yang et al. (94)	dual-attention U-Net	Simens Acuson S2000	ABUS	188	internal center dataset: DSC= 84.97% 95HD=6.22mm external validation set: DSC= 80.79% 95HD=9.82mm	The study proposed a dual-attention improved U-Net with interlayer information fusion to leverage sequential ABVS frames for tumor segmentation	Multi-center study; External validation	lesions segmentation
2024	Malekmohammadi et al. (95)	2D attentive UNet	Simens Acuson S2000	ABUS	43	DSC=0.8582, JI=0.751, PRE=0.8716, REC=0.8452, HD95 = 5.233mm	The study proposed an enhanced attentive U-Net with bidirectional ConvLSTM and saliency-guided multi-scale feature fusion for tumor segmentation	Single-center study; Internal validation	lesions segmentation
2024	Li et al. (96)	DeepLab-V3	Simens Acuson S2000	ABUS	216	DSC=0.890 ± 0.152, REC=0.972 ± 0.167, PRE=0.948 ± 0.198	The study developed a two-stage deep learning framework combining automatic segmentation with DeepLab-V3 and multiview feature fusion for lesions segmentation	Multi-center study; External validation	lesions segmentation
2024	Li et al. (97)	GLGM	Simens Acuson S2000	ABUS	207	the private ABUS dataset: DSC= 75.49%, JC= 64.71%, 95HD= 22.26 mm, ASD= 8.08 mm the public dataset: DSC= 72.65%, JC= 61.75%, 95HD= 7.84 mm, ASD= 3.75 mm	The study proposed a dual-branch encoder-decoder network with global-local feature fusion and graph convolution to enhance small tumor segmentation	Multi-center study; External validation	lesions segmentation
2020	Wang et al. (77)	3D U-Net	GE Healthcare Invenia ABUS	ABUS	264	SEN=87.0%, SPE=88.0%, ACC= 87.5%, AUC= 92.2%	The study proposed a 3D U-Net with spatial attention and residual blocks for tumor classification	Single-center study; Internal validation	lesions classification
2020	Wang et al. (98)	Inception-v3	Simens Acuson S2000	ABUS	263	AUC=0.9468, SEN=88.6%, SPE=87.6%	The study proposed a multiview CNN based on modified Inception-v3 with transfer learning for classification	Single-center study; Internal validation	lesions classification
2020	Zhou et al. (87)	CMSVNet	Simens Acuson S2000	ABUS	107	AUC=0.787, ACC=0.741, F1 score=0.811	The study proposed a multi-task learning framework combining a segmentation encoder-decoder network and a multi-scale classification branch, enhanced by iterative feature refinement, to jointly improve tumor segmentation and classification	Single-center study; Internal validation	lesions classification
2021	Zhuang et al. (99)	GRUC-Net	IBUS BE3	ABUS	214	ACC=0.9286, REC= 0.8824, PRE= 0.9375, F1 score= 0.9091, AUC= 0.9721	The study proposed a modified feature extraction network combined with GRU to classify breast tumors	Single-center study; Internal validation	lesions classification
2021	Xiang et al. (100)	3D Res-CapsNet	GE Healthcare Invenia ABUS	ABUS	396	ACC=84.9%, SEN=87.2%, SPE=82.6%, AUC=0.9122	The study proposed a 3D Res-CapsNet combined with U-net segmentation to classify breast tumors	Single-center study; Internal validation	lesions classification
2022	Hejduk et al. (101)	CNN	GE Healthcare Invenia ABUS	ABUS	113	ACC=90.9%, AUC=0.91	The study developed a deep convolutional neural network with a sliding-window approach for automatic detection and classification of breast lesions	Single-center study; Internal validation	lesions classification
2022	Kim et al. (102)	DenseNet	GE Healthcare Invenia ABUS	ABUS	363	mass classification:SEN =87.75%, SPE =93.75%, AUC=0.9491 cancer classification:SEN=63.95%, SPE=61.57%, AUC=0.6802	The study developed a deep convolutional neural network with a mask branch network and template masks derived from radiology reports for classifying breast lesions	Single-center study; Internal validation	lesions classification
2023	Ding et al. (103)	MVSA-Net	IBUS BE3	ABUS	214	ACC=95.24%, AUC=99.76%	The study proposed a multi-view stereoscopic attention network with 3D localization and Transformer-based classification	Single-center study; Internal validation	lesions classification
2024	Li et al. (96)	Inception-v3	Simens Acuson S2000	ABVS	216	AUC=0.949, SEN=82.14%, SPE=95.56%	The study developed a multiview deep learning model with automatic segmentation (DeepLab-V3) and classification (Inception-v3 backbone) based on ABVS images for identifying breast cancer	Multi-center study; External validation	lesions classification

3D CNN, three-dimensionally convolutional neural network; 3D U-Net, three-dimensionally U-shaped convolutional neural network; Faster R-CNN, fast region-based convolutional neural networks; SSL-E, semi-supervised learning EfficientDet; R-CNN, region-based convolutional neural networks; DSGMFFN, deepest semantically guided multi-scale feature fusion network; MDCCM, multidomain consistency constraint model; GLGM, global and local feature interaction model combined with graph fusion; GRUC-Net, GRU Classified Network; CMSVNet, classification branch for multi-task learning network; 3D Res-CapsNet, three-dimensionally residual-capsuleneural network; MVSA-Net, multiview stereoscopic attention network; IoU, Intersection over Union; CenDis, centers of detected and ground truth cancers; FPs, false positives; SEN, sensitivity; DSC, Dice similarity coefficient; JAC, Jaccard index; HD95, 95% Hausdorff distance; MSD, mean surface distance; RMSD, residual mean square distance; CMD, center of mass distance; REC, Recall; PRE, Precision; JI, Jaccard index; AP, average precision; JC, Jaccard coefficient; ASD, average surface distance; AUC, area under the curve; ACC, accuracy.

### 5.2 Application of deep learning for ABUS radiomics

#### 5.2.1 Lesion detection

Traditional ML methods for identifying breast lesions are largely manual, whereas DL methods enable automatic tumor detection. Several studies have applied DL for breast lesion detection using HHUS. For example, Yap et al. systematically compared three DL architectures (patch-based LeNet, U-Net, and transfer learning-optimized FCN-AlexNet) with four conventional methods for breast ultrasound lesion detection. The results demonstrated that FCN-AlexNet achieved optimal performance with a True Positive Fraction of 0.98, indicating excellent lesion detection capability (104). Currently, the DL frameworks used for lesion detection in ABUS images mainly include 3D CNN, 3D U-Net, and R-CNN.

Unlike HHUS, the main challenge for lesion detection in ABUS radiomics is how to correctly process 3D images instead of 2D images (105), regarding lesion detection in ABUS, previous approaches have mainly focused on clustering the localization results of 2D slices to form the final 3D results (106, 107). Oh et al. developed a 3D breast nodule detection system combining 2D Faster R-CNN and U-Net. The method employs confidence thresholding and hierarchical clustering to reduce 2D false positives (FPs), then aggregates sequential 2D detections into 3D cuboids. Evaluation results showed 90.98% SEN (11.6 FPs/case) on Dataset A and 93.65% SEN (8.6 FPs/case) on Dataset B (82).

Wang et al. proposed an improved 3D CNN architecture for cancer detection in ABUS. The study achieved outstanding performance with 95% SEN and only 0.84 FPs per volume. This was accomplished by introducing 3D dilated convolutions to enhance multi-scale feature extraction, optimizing the training process with a hybrid loss function, and designing an adaptive threshold map to refine the cancer probability map. However, the method still has limitations, including misclassification of benign lesions, missed detection of small cancerous regions, high computational demands of the 3D network. Future research will focus on improving malignancy classification, optimizing shadow region detection, and enhancing efficiency through lightweight network design (76).

DL offers significant advantages in tumor detection, including high efficiency and automation, effectively enhancing physicians’ diagnostic workflow. However, non-tumorous regions such as vascular dilatation and glandular shadows may still lead to FPs, while the SEN for small lesions remains notably lower than that for larger tumors. These limitations indicate significant opportunities for further optimization and enhancement of the technology.

#### 5.2.2 Tumor segmentation

In addition to detection, tumor segmentation is clinically significant, as it helps to precisely delineate the boundaries of lesions (108). This segmentation forms the foundation for tumor analysis, lesion load assessment, and surgical planning (109). In early studies, mass segmentation primarily relied on manually selected features (110–112). However, manual feature selection has inherent drawbacks, including inefficiency and subjectivity. To address these issues, Kozegar et al. developed a new adaptive region growing algorithm combined with deformable model. This approach achieved a mean Dice of 0.74 ± 0.19 (110). In recent years, automatic tumor segmentation has become mainstream, with commonly used DL frameworks including U-Net, R-CNN, and V-Net.

Lei et al. developed a region-based CNN incorporating a mask scoring mechanism for automated segmentation of breast tumors in ABUS images. The method achieved a mean Dice similarity coefficient (DSC) of 82.1% ± 14.5% on the independent test set. However, its complexity requires substantial computational resources, limiting its practical applicability (85).

Segmentation architecture design relies heavily on expert knowledge. Neural architecture search automates this process. Cao et al. proposed Auto-DenseUNet, which leverages neural architecture search automates and a densely connected structure to optimize feature fusion through multi-scale aggregation nodes. A decoupled search-training strategy was introduced to balance search efficiency and model performance. On the ABUS dataset, Auto-DenseUNet achieved a mean DSC of 77.8% and demonstrated competitive performance on a cardiac MRI dataset. However, domain shift across datasets remains a challenge for further investigation (88).

Zhou et al. proposed CMANetfusion, a cross-model attention segmentation network integrating 3D Mask R-CNN with V-Net. This framework achieves tumor segmentation in ABUS images through probability map-guided feature fusion and a hybrid loss function, attaining a DSC of 64.57% on single-center data. However, the model demonstrates limitations in segmenting small tumors and lesions adjacent to nipples, while exhibiting high computational demands. Future optimizations could incorporate multi-task learning and lightweight architecture design to enhance performance (91).

DL models can automatically extract features, significantly improving segmentation efficiency while reducing manual intervention. However, current research primarily focuses on static images, leaving the dynamic segmentation capability for real-time ABUS video analysis largely unexplored. Future work should leverage self-supervised pretraining to minimize annotation dependency and develop lightweight networks to meet clinical real-time requirements.

#### 5.2.3 Classification of lesions

BI-RADS 4 lesions have a high biopsy rate, but many are benign. DL models can help distinguish benign from malignant lesions, reducing unnecessary biopsies. ABUS generates 3D images, leveraging multi-view information for improved classification. However, ABUS imaging features challenge lesion classification due to pixel and grayscale limitations. Precise lesion localization is crucial, especially boundary delineation. Current methods for interpreting ABUS images are limited in stereoscopic boundary localization and feature extraction, and do not effectively utilize long-range features or attention mechanisms. Improvements are needed to fully exploit stereoscopic information and features from different views to enhance diagnostic accuracy.

Zhuang et al. proposed a tumor classification method for ABUS images called the Classifying-ABUS Architecture. This method extracts the image of interest (IOIs) and ROI employing a Shared Extracting Feature Network combining VGG16 and a novel Shallowly Dilated Convolutional Branch Network to extract both general and ultrasound-specific features. Then employs GRU Classification Network to integrate sequence features for classification. The experimental results demonstrate that the proposed method achieved a classification ACC of 92.86% for the test set (99). However, current methods still face challenges in capturing long-range spatial relationships and sophisticated feature patterns in ultrasound imaging.

Ding et al. proposed MVSA-Net, a two-stage classification method for breast ultrasound images. MVSA-Net consists of a stereo localization unit and a classification unit. The stereo localization unit uses a stereo attention module and segmentation output design to accurately locate the tumor region. The classification unit then uses a transformer network to classify the tumor as benign or malignant. This approach achieved an ACC of 95.24% with an AUC of 99.76%, significantly improving classification speed and efficiency. Key contributions include the module’s focus on the tumor’s edge regions and the transformer network’s global attention mechanism, which enables MVSA-Net to capture long-range feature dependencies (103).

A common challenge in classification models is the imbalance between malignant and benign samples, with malignant cases usually being much fewer than benign ones. This imbalance can negatively affect model training. Also, when working with small datasets, models tend to overfit the training data. To address these issues, future studies could employ data augmentation techniques to increase sample diversity and enhance model performance.

## 6 Discussion

The combination of AI and ABUS radiomics has demonstrated multifaceted clinical application value in BC diagnosis and treatment assessment, significantly improving tumor detection and diagnostic accuracy while effectively predicting tumor molecular marker expression levels and assessing ALND risk. Current research shows that the multimodal joint modeling of ABUS with B-ultrasound and SE can achieve complementary advantages: ultrasound compensates for ABUS’s limitations in detecting peripheral lesions, poor imaging performance in large breasts, and restricted evaluation of axillary and nipple regions through its flexibility (113), while SE further enriches model features by quantifying tissue stiffness as an important biological characteristic (40). However, existing studies are mostly limited to the joint analysis of ABUS with B-ultrasound and SE, with relatively insufficient research on cross-modal fusion with other imaging techniques such as CEU, MRI, and CT. ABUS radiomics is increasingly being combined with key pathomics elements like histological grading and immunohistochemical markers, as well as clinomics parameters including survival data and hematological features, representing a growing research priority. By establishing quantitative correlations between imaging features and pathological microenvironment and molecular subtypes, we can further reveal the biological basis of ABUS signs. Currently, there remains a significant gap in key technology research for DL-based multimodal data fusion of ABUS, which will become an important direction for future research.

However, it is noteworthy that while these advanced technologies enable precise diagnosis, they still face several key challenges in clinical practice. First, existing studies generally suffer from insufficient external validation data. In the process of promoting data diversity, it is crucial to ensure the prompt implementation of standardized protocols and enhance the security of multi-center collaboration through strengthened privacy data measures. Additionally, different medical institutions may use different models of ABUS equipment, and variations in image resolution, scanning parameters, and coronal reconstruction algorithms among different manufacturers’ ABUS devices exist. Most models are only trained on single-device data, so the generalization capability of models across different ABUS devices still requires validation. Beyond equipment differences, the lack of standardized protocols may introduce data heterogeneity due to variations in operator experience. Therefore, strict adherence to the manufacturer’s scanning guidelines is essential when using ABUS. Furthermore, as a core component of medical big data, medical imaging data is subject to strict data sharing regulations due to its storage standards, clinical application scenarios, and privacy sensitivity (114, 115). Current privacy protection technologies mainly focus on data de-identification and differential privacy (114, 116), but their privacy protection effectiveness still needs evaluation. McMahan et al. proposed federated learning (FL), a distributed ML framework (115). This framework coordinates multi-center collaboration through a central server: each hospital trains models using local data and only uploads parameter updates to the central server to generate a global model, which is then returned to each institution. This approach enables the mining of multi-center data value while protecting local data. Current research has confirmed its feasibility in ultrasound image pre-training (117), providing new ideas for ABUS privacy data research.

While addressing challenges of data privacy protection and model generalization capability, AI models face another critical bottleneck in clinical applications - the problem of insufficient interpretability. So-called “black box” models refer to algorithmic systems that lack transparency in their internal decision-making mechanisms (118). Such models often focus too much on the mapping relationship between input and output during development while neglecting the visualization of decision processes, making it difficult for clinicians to understand the model’s specific reasoning logic. This comprehension gap easily leads to clinicians’ distrust of AI diagnostic results. More importantly, even if the model achieves high prediction accuracy, its output may lack clinical interpretability, reducing the operability of prediction conclusions (117). Therefore, promoting Explainable Artificial Intelligence (XAI) appears particularly important. XAI aims to make the decision-making process of AI applications transparent not only to domain experts or data scientists but also to clinicians unfamiliar with AI complexity through specific methods and technologies (119). Current XAI research mainly focuses on six methods: Feature-oriented methods, global methods, concept models, surrogate models, local pixel-based methods, and human-centric methods. Feature-oriented methods related to radiomics can quantify features’ contribution to model prediction; Concept Models can translate models into concepts understandable by physicians; Surrogate Models can replace complex model predictions by constructing simple models, providing reliable basis for personalized medicine; Local, Pixel-based Methods can display which pixels in the input image are most critical for model prediction; Human-centric Methods mainly emphasize explaining models from cognitive habits (120). Applying these XAI methods to ABUS radiomics analysis in the future will significantly improve model interpretability and clinical practicality.

In traditional ML and DL classification tasks, models typically require large amounts of manually annotated data for training. The data annotation process is not only time-consuming and expensive but may also introduce noise due to labeling errors, affecting model performance. In contrast, self-supervised learning (SSL) generates supervisory signals automatically from unlabeled data (such as predicting image rotation angles, filling in missing parts, or contrastive learning), significantly reducing dependence on manual annotation and thereby decreasing data annotation costs and complexity. SSL effectively solves the problems of high annotation costs, insufficient generalization capability, and low computational efficiency by utilizing the intrinsic structure of unlabeled data to generate supervisory signals. In the future, this method is expected to be applied to unannotated datasets such as ABUS, further improving the efficiency and accuracy of medical image analysis (121).

AI, with its exceptional analytical capabilities, has become an important technological tool in ABUS radiomics research. However, challenges such as insufficient model generalization, data privacy protection, and model interpretability still need to be addressed. In the future, overcoming these challenges will require close collaboration among multidisciplinary teams. On one hand, standardized ABUS imaging databases need to be established, and more prospective clinical studies should be conducted to validate the practical value of AI-assisted diagnostic systems. Simultaneously, intuitive visualization models need to be developed to help clinicians understand AI decision-making processes.

## Glossary

ABUS: Automatic breast ultrasound
ABVS: Automated breast volume scanner
ACC: Accuracy
ADCs: Antibody-drug conjugates
AI: Artificial intelligence
ALND: Axillary lymph node metastases
AP: Axial plane
AUC: Area Under the Curve
BC: Breast cancer
CAD: Computer-aided
CEU: Contrast-enhanced ultrasound
CNN: Convolutional Neural Network
CP: Coronal plane
DBT: Digital breast tomosynthesis
DL: Deep learning
DSC: Dice similarity coefficient
FDA: Food And Drug Administration
FL: Federated learning
FPs: False positives
GBDT: Gradient Boosted Decision Trees
HER2: Human epidermal growth factor receptor 2
HHUS: Handheld ultrasound
IARC: International Agency for Research on Cancer
IOIs: Image of interest
LASSO: Least Absolute Shrinkage and Selection Operator
LNs: Lymph nodes
LR: Logistic Regression
MG: Mammography
ML: Machine learning
MRI: Magnetic resonance imaging
NAT: Neoadjuvant therapy
R-CNN: Region-based convolutional neural network
PRE: Precision
ROIs: Regions of interest
SE: Strain elastography
SEN: Sensitivity
SPE: Specificity
SSL: Self-supervised learning
SVM: Support Vector Machine
TKIs: Tyrosine kinase inhibitors
XAI: Explainable Artificial Intelligence
XGBoost: Extreme Gradient Boosting
